# Supplementary material for: The nose knows: Thermal responses to active psychological stressors
Source: PLoS One. 2026 Jan 8;21(1):e0338108. doi: 10.1371/journal.pone.0338108 (PMC12782435; doi:10.1371/journal.pone.0338108)
Supplement: S1 File — (DOCX) [file pone.0338108.s001.docx]

**Supplementary material a.** Script of the interactions with participants thought the TSST.

**Face Value Protocol (University of Sussex, Comparative Cognition Group):**

*"Hello and welcome. Thank you for participating today. We are interested in examining how individuals respond to stressful situations, particularly focusing on heart rate variability and thermal changes in facial temperature during and after the stressor. First, we have a couple quick questions to make sure you are still able to take part. Have you consumed any vasoactive substances today? Have you had any caffeinated or alcoholic beverages in the last three hours? Before we begin the main part of the study, we would like you to complete brief surveys on our tablet. These surveys will help us gather some background information and measure your current state before we start the task. Please take your time and let me know if you have any questions about the surveys. Here is your participant number, you will need it for the surveys. Answer as many questions as you can but do skip a question if you do not feel comfortable.”*

**Pre task baseline**

*"We will start with five minutes of white noise. We are now going to start recording on our cameras. Can you please hold this paper in front of the camera so that we can later confirm that your video results can be linked with your answers in the surveys. Here are the headphones; is the volume okay? Please relax, close your eyes, and we’ll return in 5 minutes."*

**Speech Task:**

*"In a moment, you'll deliver a short speech. You will be asked to take over the role of a job applicant who is invited for a personal interview with a "selection committee”. You will have 3 minutes to prepare a speech, and then you’ll present it to us, the committee, for 5 minutes. You will have to introduce yourself and convince us that you are the perfect applicant for the job. You can start by presenting the job you are applying for. You can choose the job that you want. We have been specially trained to monitor non-verbal behaviour. You will be filmed, and your voice will be recorded by a camera throughout the session for later analysis of your performance. Voice frequency analysis of non-verbal behaviour and video analysis of your performance will be performed. We will also be measuring your heart rate using a monitor to track how your body responds during the task. After your speech, we might ask you a few follow-up questions and will present another task later."*

*Prepared questions if the participant finishes before the 5 minutes are over: ‘’What are your personal strengths? What are your major shortcomings? What do you think about teamwork? What do your boss/family/colleagues think about you? Why? Do you have enemies? Why? Talk about a time you had to deal with conflict at workplace and how you’ve gone through it? Talk about your previous job experience.’’*

**Arithmetic Task**

*“Now, you will complete an arithmetic task. Starting from 2023, subtract 17 until you reach zero, as quickly and accurately as possible. If you make a mistake, you will have to start again.”*

*Each time the participant fails: “Stop, mistake, start over at 2023, please.”*

**Post tasks recovery**

*“Thank you for completing the tasks. You can take a moment to relax now. We will give you a short break by listening to the white noise for five minutes. Then will then ask you to fill a final survey. I’d like to now disclose you we have not and will not judge your performance. We don’t keep any audio recording of the experiment. This task was specifically designed to make you feel stressed. It is normal if you felt like the speech and the arithmetic tasks were hard to perform. Here are the headphones for the white nose. Is the volume ok? Please relax, close your eyes and stay still. We will come back in 5 minutes.” After the recovery period: “Now that you've had a brief rest, we would like you to complete a final survey to assess how you’re feeling after the task.”*

**Debrief**

*“Once again, thank you for your participation. We’d like to take a few moments to explain the purpose of this study in more detail. The aim of this study is to examine how individuals respond to acute stress. Specifically, we were assessing your heart rate variability, which helps us understand how your body physically responds to stress, as well as changes in the thermal temperature of your face throughout the session. These measures allow us to track how your body responds during the stressful situation. We also investigate how long it takes for your physiological state to return to normal afterward. The task you participated in was designed to create a moderate level of stress, and the filming and heart monitoring were tools used to measure these responses. As we said before, no voice frequency or video analysis will be performed unless explicitly stated otherwise. We are not going to judge your performance at all. We don’t keep any audio recording of the experiment. This task was specifically made to make you feel stressed. It is totally normal if you felt like the speech and the arithmetic tasks were hard to perform. Do you have any questions about the study or how the data will be used? Please don’t discuss too much about what you did during the experiment with people you know that want to take part in our study, just so we can recreate the same experience for them. If you have any concerns or would like more information about the study, feel free to reach out to us at any time using the contact information we gave you. Have a great day!”*
