# Supplementary material for: The nose knows: Thermal responses to active psychological stressors
Source: PLoS One. 2026 Jan 8;21(1):e0338108. doi: 10.1371/journal.pone.0338108 (PMC12782435; doi:10.1371/journal.pone.0338108)
Supplement: S1 Table — (DOCX) [file pone.0338108.s002.docx]

**S1 Table**

Method reliability tests, performed on 17% on the participants, were showing a strong degree of agreement across the two methods (Spearman’s correlation)

| **Thermal Variables** | **r** | **p value** |
| --- | --- | --- |
| Maximum Baseline Temperature | 1 | .017 |
| Minimum Temperature During Speech Task | 1 | .017 |
| Temperature Drop During Speech Task | 1 | .017 |
| Minimum Temperature During Arithmetic Task | 1 | .017 |
| Temperature Drop During Arithmetic Task | 0.6 | .35 |
| Temperature After 5 Minutes Recovery | 0.9 | .017 |
