# Supplementary material for: The nose knows: Thermal responses to active psychological stressors
Source: PLoS One. 2026 Jan 8;21(1):e0338108. doi: 10.1371/journal.pone.0338108 (PMC12782435; doi:10.1371/journal.pone.0338108)
Supplement: S2 File — (DOCX) [file pone.0338108.s004.docx]

**S2 Supporting Information. Ambient temperature and age effect**

**(*text explains S1 Fig)**

Ambient temperature and age effect:

Spearman’s correlations reveal several positive associations between nasal thermal responses and ambient temperature: Maximum Baseline Temperature (ρ = 0.66, p < .001), Minimum Temperature During Speech Task (ρ = 0.61, p < .001) and Minimum Temperature During Arithmetic Task (ρ = 0.54, p = .002), Temperature After 5 Minutes Recovery (ρ = 0.66, p < .001).

In addition, age was negatively associated with Maximum Baseline Temperature (ρ = -0.56, p = .002), Minimum Temperature During Speech Task (ρ = -0.42, p =.02), Minimum Temperature During Arithmetic Task (ρ = -0.56, p = .002), Temperature After 5 Minutes Recovery (ρ = -0,54, p = .003), and Thermal Recovery Rate at 5 Minutes (ρ = -0.40, p = .03).

Age was shown association with some psychological score. Age was negatively correlated with STICSA - Total Score (ρ = -0.47, p = .01) and STICSA - Cognitive Subscore (ρ = -0.568 p = .001). No significative association were found between age and STICSA - Somatic Subscore and PSS - Total Score.
