# Supplementary material for: The nose knows: Thermal responses to active psychological stressors
Source: PLoS One. 2026 Jan 8;21(1):e0338108. doi: 10.1371/journal.pone.0338108 (PMC12782435; doi:10.1371/journal.pone.0338108)
Supplement: S2 Table — (DOCX) [file pone.0338108.s005.docx]

**Table 2.** Descriptive statistics for facial skin temperature measures across all participants.

| Participant Number | Maximum Baseline Temperature | Minimum Temperature - Speech Task | Drop Temperature - Speech Task | Minimum Temperature - Arithmetic Task | Drop Temperature - Arithmetic Task | Temperature after 5min Recovery |
| --- | --- | --- | --- | --- | --- | --- |
| S001 | 31.36 | 29.82 | 1.55 | 30.14 | 1.23 | 32.2 |
| S002 | 36.21 | 33.75 | 2.47 | 35.22 | 1 | 35.71 |
| S003 | 34.14 | 30.34 | 3.8 | 30.12 | 4.02 | 29.95 |
| S004 | 34.32 | 29.86 | 4.45 | 31.28 | 3.04 | 34.69 |
| S005 | 35.33 | 31.95 | 3.38 | 32.4 | 2.93 | 34.54 |
| S006 | 35.58 | 29.87 | 5.71 | 31.27 | 4.32 | 36.35 |
| S007 | 33.01 | 30.88 | 2.13 | 30.58 | 2.43 | 31.09 |
| S008 | 35.89 | 30.02 | 5.87 | 32.03 | 3.86 | 36.3 |
| S009 | 36.18 | 34.6 | 1.58 | 34.13 | 2.05 | 35.83 |
| S010 | 36.36 | 35.11 | 1.25 | 35.47 | 0.89 | 36.02 |
| S011 | 35.28 | 33.2 | 2.08 | 33.8 | 1.47 | 34.45 |
| S012 | 35.47 | 33.14 | 2.33 | 33.58 | 1.89 | 35.34 |
| S013 | 32.21 | 29.8 | 2.41 | 29.74 | 2.47 | 30.02 |
| S014 | 36.01 | 30.68 | 5.33 | 32.63 | 3.38 | 36.15 |
| S016 | 36.71 | 33.89 | 2.82 | 34.72 | 2 | 37.05 |
| S017 | 36.38 | 34.62 | 1.76 | 34.5 | 1.88 | 36.41 |
| S018 | 35.8 | 30.74 | 5.06 | 33.92 | 1.88 | 35.22 |
| S019 | 30.62 | 28.62 | 1.99 | 28.74 | 1.88 | 28.9 |
| S020 | 35.74 | 33.91 | 1.84 | 34.66 | 1.09 | 36.02 |
| S021 | 36.19 | 31.05 | 5.14 | 30.93 | 5.26 | 36.55 |
| S022 | 36.2 | 34.44 | 1.76 | 34.43 | 1.77 | 35.72 |
| S023 | 34.82 | 30.86 | 3.96 | 30.83 | 3.99 | 32.66 |
| S024 | 34.67 | 28.18 | 6.49 | 32.06 | 2.61 | 29.56 |
| S025 | 35.49 | 29.88 | 5.62 | 29.81 | 5.68 | 33.21 |
| S026 | 32 | 28.22 | 3.79 | 29.62 | 2.38 | 29.25 |
| S027 | 35.55 | 32.83 | 2.71 | 34.34 | 1.21 | 35.75 |
| S028 | 29.65 | 27.61 | 2.04 | 28.84 | 0.81 | 27.33 |
| S029 | 31.84 | 27.61 | 4.23 | 27.07 | 4.77 | 27.27 |
| S033 | 28.93 | 26.88 | 2.05 | 27.96 | 0.97 | 27.81 |
