# Supplementary material for: The nose knows: Thermal responses to active psychological stressors
Source: PLoS One. 2026 Jan 8;21(1):e0338108. doi: 10.1371/journal.pone.0338108 (PMC12782435; doi:10.1371/journal.pone.0338108)
Supplement: S3 Table — (DOCX) [file pone.0338108.s006.docx]

**S3 Table** Partial spearman correlations —with age and ambient temperature as control—between nasal skin temperature and psychological scores.

| **Thermal Variables** | **Predictor** | **ρ** | **p value** |
| --- | --- | --- | --- |
| Maximum Baseline Temperature | PSS – Total Score | -0.104 | 0.6071 |
| Maximum Baseline Temperature | STICSA – Total Score | 0.034 | 0.8668 |
| Maximum Baseline Temperature | STICSA – Cognitive Anxiety Subscore | 0.084 | 0.6776 |
| Maximum Baseline Temperature | STICSA – Somatic Anxiety Subscore | -0.057 | 0.7778 |
| Temperature After 5 Minutes Recovery | PSS – Total Score | 0.021 | 0.9175 |
| Temperature After 5 Minutes Recovery | STICSA – Total Score | 0.094 | 0.6413 |
| Temperature After 5 Minutes Recovery | STICSA – Cognitive Anxiety Subscore | 0.085 | 0.675 |
| Temperature After 5 Minutes Recovery | STICSA – Somatic Anxiety Subscore | 0.079 | 0.6948 |
| Minimum Temperature During Speech Task | PSS – Total Score | -0.043 | 0.8307 |
| Minimum Temperature During Speech Task | STICSA – Total Score | -0.168 | 0.4025 |
| Minimum Temperature During Speech Task | STICSA – Cognitive Anxiety Subscore | 0.021 | 0.9167 |
| Minimum Temperature During Speech Task | STICSA – Somatic Anxiety Subscore | -0.27 | 0.1732 |
| Minimum Temperature During Arithmetic Task | PSS – Total Score | -0.036 | 0.8593 |
| Minimum Temperature During Arithmetic Task | STICSA – Total Score | -0.003 | 0.988 |
| Minimum Temperature During Arithmetic Task | STICSA – Cognitive Anxiety Subscore | 0.138 | 0.4928 |
| Minimum Temperature During Arithmetic Task | STICSA – Somatic Anxiety Subscore | -0.119 | 0.5559 |
| Thermal Recovery Rate at 5 Minutes | PSS – Total Score | 0.147 | 0.4643 |
| Thermal Recovery Rate at 5 Minutes | STICSA – Total Score | 0.182 | 0.3635 |
| Thermal Recovery Rate at 5 Minutes | STICSA – Cognitive Anxiety Subscore | 0.078 | 0.6987 |
| Thermal Recovery Rate at 5 Minutes | STICSA – Somatic Anxiety Subscore | 0.242 | 0.2229 |
| Temperature Drop During Speech Task | PSS – Total Score | 0.127 | 0.5291 |
| Temperature Drop During Speech Task | STICSA – Total Score | 0.464 | 0.0147 |
| Temperature Drop During Speech Task | STICSA – Cognitive Anxiety Subscore | 0.241 | 0.2265 |
| Temperature Drop During Speech Task | STICSA – Somatic Anxiety Subscore | 0.469 | 0.0137 |
| Temperature Drop During Arithmetic Task | PSS – Total Score | -0.003 | 0.989 |
| Temperature Drop During Arithmetic Task | STICSA – Total Score | 0.211 | 0.2906 |
| Temperature Drop During Arithmetic Task | STICSA – Cognitive Anxiety Subscore | 0.067 | 0.7394 |
| Temperature Drop During Arithmetic Task | STICSA – Somatic Anxiety Subscore | 0.203 | 0.31 |
