# Supplementary material for: The nose knows: Thermal responses to active psychological stressors
Source: PLoS One. 2026 Jan 8;21(1):e0338108. doi: 10.1371/journal.pone.0338108 (PMC12782435; doi:10.1371/journal.pone.0338108)
Supplement: S3 File — (DOCX) [file pone.0338108.s007.docx]

**S3 Supporting Information.** Calculations to derive variables for the analysis, using the thermal samples from IRT video collection.

Baseline – protocol for collection

- Initial Temperature: Average temperature of the 10 first seconds of the experiment
- Maximum Baseline Temperature: Maximum temperature reach during pre-experimental baseline (1st white noise) (average 10s around the maximum value)

Speech and Arithmetic conditions – protocol for collection

- Minimum Temperature During Speech/Arithmetic Task: Lowest temperature reach in the condition task (average 10s around the minimum value)
- Time to Minimum Temperature During Speech/Arithmetic Task: Time to reach Minimum Temperature During Speech/Arithmetic Task after the beginning of task (sec)
- Temperature Drop During Speech Task: Maximum number of degrees the individual lost during task from the Maximum Baseline Temperature

Recovery – protocol for collection

- Temperature After 5 Minutes Recovery: Temperature 5min after the beginning of the post-experimental recovery (2^nd^ white noise) (average 10s around the 5min value)
- Thermal Recovery Rate at 5 Minutes: Recovery rate 5min after the beginning of post-experimental recovery (2nd white noise) (in %) calculate with: Thermal Recovery Rate at 5 Minutes = (Temperature After 5 Minutes Recovery - Minimum Temperature) * 100 / (Maximum Baseline Temperature - Minimum Temperature) (with Minimum Temperature being the lowest temperature between Minimum Temperature During Speech or Arithmetic Task.
