# Supplementary material for: The nose knows: Thermal responses to active psychological stressors
Source: PLoS One. 2026 Jan 8;21(1):e0338108. doi: 10.1371/journal.pone.0338108 (PMC12782435; doi:10.1371/journal.pone.0338108)
Supplement: S4 File — (DOCX) [file pone.0338108.s008.docx]

**S4 Supporting Information. Sex and Perceived Stress**

**(*text explains S2-S3 Figs)**

T-test was performed to see that mean perceived stress scores (PSS) significantly differed (p = .03) between males (m = 12.5 ± 5.48), and females (m = 17.42 ± 5.06). The distribution of participants’ perception of the most stressful task differed significantly by sex (χ² = 13.78, p < .001), with 94.74% of females identifying the arithmetic task and 80.00% of males selecting the speech task as most stressful.
