# Supplementary material for: The nose knows: Thermal responses to active psychological stressors
Source: PLoS One. 2026 Jan 8;21(1):e0338108. doi: 10.1371/journal.pone.0338108 (PMC12782435; doi:10.1371/journal.pone.0338108)
Supplement: S5 File — (DOCX) [file pone.0338108.s011.docx]

**S5 Supporting Information**

**(*text explains S4 Fig)**

Spearman’s correlations were performed to investigate correlation in-between psychological tests. The PSS – Total Score showed positive correlations with the STICSA – Total Score (r = 0.51, p < .001) and the STICSA – Somatic Subscore (r = 0.53, p < .001), and a moderate positive correlation with the STICSA – Cognitive Subscore (r = 0.4, p < .001).
