# Supplementary material for: The nose knows: Thermal responses to active psychological stressors
Source: PLoS One. 2026 Jan 8;21(1):e0338108. doi: 10.1371/journal.pone.0338108 (PMC12782435; doi:10.1371/journal.pone.0338108)
Supplement: Attachment — Submitted filename: Response to Reviewers.docx [file pone.0338108.s014.docx]

**Response to Journal and Editor Feedback**:

We confirm that: (1) our manuscript meets journal requirements regarding file naming and style (2) data resides in OSF: <https://osf.io/rk65p/overview?view_only=5ea26cdc2b5945afb388941444cde258>, (3) Figure 1 has been created using PowerPoint basic shapes (circles, squares, lines) and textboxes. Basic geometric shapes are general domain and not eligible for copyright protection, (4) Supporting Information file names and within-text citations conform to PLOS ONE guidelines and are also listed at the end of the manuscript with titles (corrections highlighted in pink in the marked-up manuscript file), and (5/6) our reference list is complete and correct. No retracted papers are cited, and all references are current, complete, and formatted according to PLOS ONE’s style requirements.

**Response to Reviewer 1:**

No specific edits have been requested by the Reviewer. However, we would like to thank Reviewer 1 for noting the real-world, translational capabilities of the methodology across a range of fields, creating a game-changing opportunity for stress detection research in both verbal and non-verbal populations.

**Response to Reviewer 2**:

Reader Experience:

Throughout the manuscript, the Reviewer has requested a general read for text smoothness and punctuation (e.g. adding Oxford commas for lists) to facilitate reader experience.

*We thank Reviewer 2 for this feedback and have also reviewed the manuscript text and added appropriate use of the Oxford comma to ensure readability.* The typographical and grammatical errors are listed below followed by the amendment in *italics*. Amendments are highlighted in blue in the marked-up manuscript.

Line 88: suggest ‘these more objective stress measures’- *(added ‘more’)*
Line 122: suggest ‘stress induced by using fear’ *(added ‘by)*

Line 134: typo ‘decreases are directly associated’ *(replaced ‘is’ with ‘are’)*
Line 150: typo ‘a relatively low sample rate’ *(replaced ‘rates’ with ‘rate’)*
Line 166: typo ‘meat was available as compared to figs’ *(added ‘as’)*
Line 170: there’s something about the grammar here (sensorily vigilant...? show sensory vigilance...?). (*edited* *text* *to read ‘increasing sensory vigilance’)*
Line 172: typo, -which (edit to comma) *(replaced ‘-which’ with ‘,’)*

Line 194: typo ‘females’ *(replaced ‘female’ with ‘females’)*

Line 265: typo ’to systematically induce..’ *(replaced ‘inducing’ with ‘induce’)*
Line 304: typo ‘were sampled’ *(replaced ‘where’ with ‘were)*
Line 308: typo ‘based on this sampling protocol’ *(inserted ‘this’ between ‘on’ and ‘sampling’)*

Line 475: typo ‘individual baselines’ *(replaced ‘baseline’ with ‘baselines’)*
Line 481: typo ‘participants’ *(replaced ‘participant’ with ‘participants’)*

Line 521: there’s an error in sentence structure *(inserted ‘the’ between ‘or’ and ‘STICSA’)*

Manuscript Section Comments:

Abstract:

Some technical terms aren’t immediately clear to the naive/general reader (e.g., I’m not sure what a ‘contact measure’ is in this context, why is ‘non-contact’ advantageous). Might be useful to clarify. Line 42,44, etc:

*We thank Reviewer #2 for this observation. We have altered ‘contact’ and ‘non-contact’ to ‘physically invasive’ and physically ‘non-invasive’ for clarity in the abstract (lines 43 and 45). We have also added ‘physical’ before ‘contact’ in the Introduction, Line 91).*

Introduction

There is a general framing of stress as a negative experience - whereas it can be both negatively and positively valanced (e.g., excitement, arousal). I wonder if it’s useful to briefly address this somewhere, before concentrating on the negative/fear side of things.

*We appreciate this insightful point and agree that stress can have positive or negative valance. We have made several edits to address this point. We have added two sentences to the start of the abstract (Lines 40-41): “Stress is an essential component of our lives. It helps to us to stay alert, motivated, and adapt to new and challenging situations. However, it is also a leading cause of poor mental wellbeing”. We have added the text ‘informing factors such as valence’ to line 74. Additionally, we have extended our discussion on this point (Lines 549-556): “Moreover, it is not yet clear if IRT can distinguish the affective valence of a stressor. In this study we focused on social stress which is often considered negative. But the act itself is neither positively nor negatively valanced, rather it is the individual’s interpretation that associates affect. This is because arousal sits at the core of a single stress system, void of valence. How stress is interpreted as either positive or negative affect, through our physiological and psychological experiences, requires further consideration, particularly if we aim to apply this method to non-verbal populations like infants and non-human great apes.”*

Methods
It might be helpful to have some brief details on recruitment that clarify what the participants knew about the test and reasons for testing (given the white coat effect mentioned earlier in the intro may well extend to participation in a study).

*We thank the Reviewer for requesting omitted details about the recruitment process. Participants were recruited using digital and paper flyers around the university and through the School of Psychology’s research participation system. They were informed that the study involved being filmed with a thermal camera during a stress-inducing task but were given no further details on the type of task(s) they would complete or any specific procedures to avoid expectation effects. This information has been added to lines 207-210). While participants may have experienced ‘white coat syndrome’ purely by attending a psychology experiment investigating stress, the room habituation period and white noise relaxation prior to the experimental stress conditions were developed and implemented to ensure that participants relaxed to their own baseline prior to stress induction.*

Line 260: in what ways were the camera settings adjusted and why (was this to set a different baseline?, window of sensitivity?). I’m not an expert, so it would be useful for replication to know more, and to have a sense of why this was needed (rather than controlling for variation in a statistical model for example).

*The following text was added (lines 266-271) to describe the and justify the camera settings during the data collection: Camera settings for ambient temperature and humidity (as measured by a thermometer and hygrometer) were set at the start of the experiment. They were rechecked following the room habituation period to ensure accurate thermal readings were acquired. These factors influence how infrared energy travels from the target to the camera. Without correction, changes in ambient temperature and humidity can distort the temperature measurements.*

Discussion

Lines 483-486: can the authors draw any parallels with other physiological processes here that would connect to the wider literature on human body processes/health e.g., time to heart rate baseline indicating cardiac fitness, or hormonal changes in response to stress, etc. (I see it’s briefly brought in later (~line 527) - but this could be expanded on/introduced here).

*We thank the Reviewer for enquiring further about parallels with other physiological processes for the bi-directional change in nasal temperature. We intentionally did not conduct contact markers of stress or relaxation in the current study; however, we have added the following text for clarity (lines 494-497): “We intentionally did not conduct contact measures of stress in the present study. However, we predict that biological measures like heart rate, blood pressure, and stress hormones (e.g. cortisol) would rise associated with stress induction and return to baseline post stress, while heart rate variability would rise in association with a body returning to rest.”*

Line 496: I’d typically refer to a group this small as ‘for the participants’ rather than ‘population’

*Thank you for this comment. We have replaced ‘population’ with ‘participants’ (Line 502).*

Line 500-506. ‘Ancient man’ is a slightly odd/outdated term and I’d suggest an alternative. But you could just refer to ‘primates’ in general (given that we have no direct evidence of whether and how our ancestors used violence in response to social standing). Could you provide a little more detail or support for the idea that ‘social damage can result in group ostracization’ - I don’t doubt that it may in some species in some circumstances, but I’m not sure how much actual evidence we have of this - nor how generalisable that is across primates (and in cases the opposite has been shown).

*We appreciate this point. The text “across primates” has been substituted for “ancient man” (Line 510). Regarding social damage resulting in group ostracization, there is quite a large body of research across disciplines (from laboratory games to neurological evidence to modern social contexts) that support the evolutionary theory. We have added two citations to the text on lines 514-15 “and still plays out in modern social settings [87-88]: [87] Williams KD. Ostracism. Annu Rev Psychol. 2007; 58:425-52. doi: 10.1146/annurev.psych.58.110405.085641. PMID: 16968209; [88] Zhang, Xuanyi. (2023). Humans Reputational Concern: How the Reputation-Based Mechanisms of Social Evolution May Be Leveraged in the Modern Context of Social Media. Lecture Notes in Education Psychology and Public Media. 7. 580-583. 10.54254/2753-7048/7/2022954.*

Line 531-543: while the sample was sufficiently powered I assume that it was systematically biased in other ways in terms of trying to describe ’human’ level physiological responses? These may be particularly important to consider where there are suggestions being made about ‘typical’ baselines/responses/therapeutic use

*This is a very apt point raised by the Reviewer. We have added the following caveat regarding our findings: “While our participant group is relatively small and will undoubtedly possess inherent biases…” (lines 562-563).*

Line 555: there is a teaser here about implications for positive conservation action, that would be interesting to unpack (I can more clearly see the welfare implications for captive living animals, but these could be clarified too?).

*We thank the reviewer for highlighting this point. We were uncertain if this was an appropriate topic to unpack in the current study, but it is the direction we are heading with the research, so we would be delighted to add a few lines about our ambitions. The following text has been added (lines 570-574), “For example, sanctuaries are mainly limited to observed, non-verbal behaviour to make decisions about ape introductions, groupings and within-species adoptions. Because all primates are prone to behavioural masking, the stress information provided via IRT may provide valuable psychological wellbeing information, beyond the mask, helping to increase the success rates of these rehabilitation activities.”*

Line 560: as above I think it would be particularly important to note the need for extension to different populations, especially where any ‘human’ norm, or species comparison is being made.

*We wholeheartedly agree with this comment and have added the following text: “It is particularly important to extend to different populations, especially where any ‘human’ norm, or species comparison is being made. Moreover, there is a need for cross-population validation and species-specific calibration before broader comparisons can be drawn.” to lines 579-582.*

Figure 4: the box plots with jitter are very nice, but perhaps it would be useful - given this is a within subjects test, to show the slopes of the lines between the points for each participant. It’s clear there’s a lot of between subject variation - but it would be nice to know if this extended to the slope between conditions as well as within them. This may be too small a sample to do so statistically - but a visualisation of the data would be a nice addition.

*We appreciate the reviewer’s thoughtful suggestion. We feel that adding the slopes of the lines between the conditions for each participant will make for very busy viewing, adding 87 lines to the figure. We are concerned the additional graphics will detract from the group result. However, we provide a supplementary table (S2 Table) that reports the change in temperature between the conditions for each participant so that readers can clearly see the pattern of drop for all participants between baseline and the induced stress conditions.*
